# pH-responsive targeted nanoparticles release ERK-inhibitor in the hypoxic zone and sensitize free gemcitabine in mutant K-Ras-addicted pancreatic cancer cells and mouse model

**DOI:** 10.1371/journal.pone.0297749

**Published:** 2024-04-30

**Authors:** Debasmita Dutta, Priyanka Ray, Archana De, Arnab Ghosh, Raj Shankar Hazra, Pratyusha Ghosh, Snigdha Banerjee, Francisco J. Diaz, Sunil P. Upadhyay, Mohiuddin Quadir, Sushanta K. Banerjee

**Affiliations:** 1 Department of Coatings and Polymeric Materials, North Dakota State University, Fargo, ND, United States of America; 2 Cancer Research Unit, VA Medical Center, Kansas City, MO, United States of America; 3 Department of Pathology and Laboratory Medicine, University of Kansas Medical Center, Kansas City, KS, United States of America; 4 Department of Biostatistics & Data Science, University of Kansas Medical Center, Kansas City, KS, United States of America; OUHSC: The University of Oklahoma Health Sciences Center, UNITED STATES

## Abstract

Therapeutic options for managing Pancreatic ductal adenocarcinoma (PDAC), one of the deadliest types of aggressive malignancies, are limited and disappointing. Therefore, despite suboptimal clinical effects, gemcitabine (GEM) remains the first-line chemotherapeutic drug in the clinic for PDAC treatment. The therapeutic limitations of GEM are primarily due to poor bioavailability and the development of chemoresistance resulting from the addiction of mutant-K-RAS/AKT/ERK signaling-mediated desmoplastic barriers with a hypoxic microenvironment. Several new therapeutic approaches, including nanoparticle-assisted drug delivery, are being investigated by us and others. This study used pH-responsive nanoparticles encapsulated ERK inhibitor (SCH772984) and surface functionalized with tumor-penetrating peptide, iRGD, to target PDAC tumors. We used a small molecule, SCH772984, to target ERK1 and ERK2 in PDAC and other cancer cells. This nanocarrier efficiently released ERKi in hypoxic and low-pH environments. We also found that the free-GEM, which is functionally weak when combined with nanoencapsulated ERKi, led to significant synergistic treatment outcomes *in vitro* and *in vivo*. In particular, the combination approaches significantly enhanced the GEM effect in PDAC growth inhibition and prolonged survival of the animals in a genetically engineered KPC (LSL-KrasG12D/+/LSL-Trp53R172H/+/Pdx-1-Cre) pancreatic cancer mouse model, which is not observed in a single therapy. Mechanistically, we anticipate that the GEM efficacy was increased as ERKi blocks desmoplasia by impairing the production of desmoplastic regulatory factors in PDAC cells and KPC mouse tumors. Therefore, 2^nd^ generation ERKi (SCH 772984)-^iRGD-pH^NPs are vital for the cellular response to GEM and denote a promising therapeutic target in PDAC with mutant K-RAS.

## Introduction

Pancreatic ductal adenocarcinoma (PDAC) is one of the most common and deadliest pancreatic neoplasms because it remains a disease with a poor prognosis [1, 2]. PDAC is the third leading cause of cancer-associated death in the United States and is expected to be the second leading cause of cancer-related deaths globally by 2030 [2]. All Surveillance, Epidemiology, and End Results (SEER) combined analysis indicated that roughly 11 percent of PDAC patients survive over five years.

Previously, our studies and other genome analyses have shown that about 95 percent of PDAC patients contain mutations in codons 12, 13, or 16 in a small GTPase protooncogene K-RAS [3–8], leading to uncontrolled activation of the K-RAS protein [9, 10]. As a result, most PDAC growth, progression, and survival are addicted to mutant K-RAS [11]. The studies in genetically engineered mouse models (GEMMs) have confirmed the tumor initiation role of oncogenic Kras^G12D^ in PDAC, which is present in more than one in three human pancreatic cancers [12, 13]. Recently, independent studies found that selective Kras^G12D^ inhibitors are efficacious in a preclinical setup [14–17]. However, targeting the Kras^G12D^ mutation through inhibitors to treat PDAC successfully is still challenging [18, 19]. Thereby, Gemcitabine (GEM), which has weak therapeutic efficacy in PDAC, is still the first-line therapy and is administered alone or in combination with platinum analog, cisplatin (GemCis) [20], nab-paclitaxel [21], capecitabine (GemCap) [22], Abraxane or FOLFIRINOX [23–25]. These drug combinations halt PDAC progression in some patients for a while, but cancer cells eventually develop drug resistance. Thus, the patients experience disease progression [26], and that could be due to the therapy’s inability to kill the pancreatic cancer stem cells (PCSCs) or tumor-initiating cells (TICs), leading to the relapse of the disease [27–29]. Furthermore, weak drug penetration into the targeted tissue through desmoplasia, a dense fibrotic stromal barrier at the core of the tumor ecosystem, is also a critical obstacle to the therapies in PDAC [30]. In addition, the studies indicated that PCSCs might be essential in stromal differentiation to form the desmoplastic barrier in the PDAC [31].

The extracellular signal-regulated kinases (ERKs), which are Mitogen-activated protein kinase cascades (MAPKs) [32, 33], contribute to the enhancement of CSC/TICs formation, cell survival, suppression of apoptosis, desmoplasia, and the disease’s progression. These kinases also block the anti-tumor immunity and GEM action in PDAC with K-RAS mutations [34–38]. Thus, ERK was initially considered a potential therapeutic target for PDAC. However, due to the high toxicity, low penetration, and bioavailability of ERK-inhibitor (ERKi) to the PDAC, the therapeutic implication was unsuccessful, and consequently, the clinical trial was discontinued [39]. Recently, we found *in vitro* and *in vivo* assays in xenograft models that toxicity and weak penetration of an ERKi inhibitor (SCH772984) and GEM can be surmounted if the ERKi and GEM are encapsulated in pH-responsive nanocarriers, with the capacity to release the encapsulated drugs into the low pH environment of cancers [40]. However, the combination encapsulation of ERKi and GEM for therapy has difficulties adjusting the doses of the two drug combinations.

Furthermore, the efficacy of a combination therapy consisting of GEM and ERKi encapsulated in nanoparticles has not been reported in the GEMM. Thus, in this study, we investigated whether encapsulated ERKi treatment promotes GEM activity via weakening desmoplasia using relevant autochthonous mouse models of PDAC. Furthermore, hypoxic niches in tumors create an acidic extracellular setting that favors tumor progression and creates GEM resistance in PDAC [41, 42]. In this study, we thus, aimed to synthesize iRGD-based pH-sensitive nanocarriers to investigate the ERKi delivery and its functional efficacy under a hypoxic environment *in vitro* and *in vivo*. We tagged the iRGD [9 amino acid cyclic peptide sequence (CRGDKGPDC)] to pegylated pH-responsive block copolymeric nanoparticles as a tumor cell surface binding and penetrating peptide for tumor-targeted drug delivery [43–45]. Our studies showed that the iRGD conjugated, targeted SCH772984 nanoformulation, combined with free GEM, may be highly effective in killing K-RAS-mutated pancreatic cancer cells due to the controlled and selective drug delivery within the hypoxic and low pH areas of desmoplastic PDAC microenvironment and reduces the production of desmoplastic regulatory proteins in PDAC cells.

## Materials and methods

### Materials

All chemicals were purchased from Sigma-Aldrich, and anhydrous solvents were obtained from VWR and EMD Millipore. A Bruker 400 MHz spectrometer was used to record 1H NMR spectra using tetrmethylsilane (TMS) as the internal standard. An attenuated total reflectance (ATR) diamond tip was used to record IR Spectra in a Thermo Scientific Nicolet 8700 FTIR instrument. The ’ number and weight average molecular weight of the synthesized polymers were characterized by gel permeation chromatographic using the GPC EcoSECsystem using polystyrene (Agilent EasiVial PS-H 4 ml) as the standard. As a mobile phase, THF was used at a flow rate of 0.35 mL/min, maintaining the column temperature at 40°C. Malvern zetasizer (Malvern ZS 90) instrument was used to measure the hydrodynamic diameter of nanoparticles. In addition, a UV-vis spectrophotometer and a fluorescent Fluoro-Log3 spectrophotometer were used to record UV-visible and fluorescence spectra of samples, respectively. The MitoProbe JC-1 assay kit was purchased from Thermo Fisher Scientific (Waltham, MA, USA).

### Cell lines and maintenance of cell lines

Pancreatic cancer cell lines, Panc-1 and MIA-PaCa-2, were procured from the American Type Culture Collection (ATCC). Panc-1 cells were cultured in high glucose DME medium, and MIA PaCa-2 cells were grown in regular DMEM (Thermo Fisher Scientific) containing 10% fetal bovine Serum (FBS) and 1% v/v Penicillin-Streptomycin. The cell lines were subcultured using enzymatic digestion of 0.25% trypsin/1mM (Thermo Fisher Scientific) upon reaching 70 percent confluency.

### Generation of autochthonous KPC pancreatic cancer mouse model

**Fig 1:**
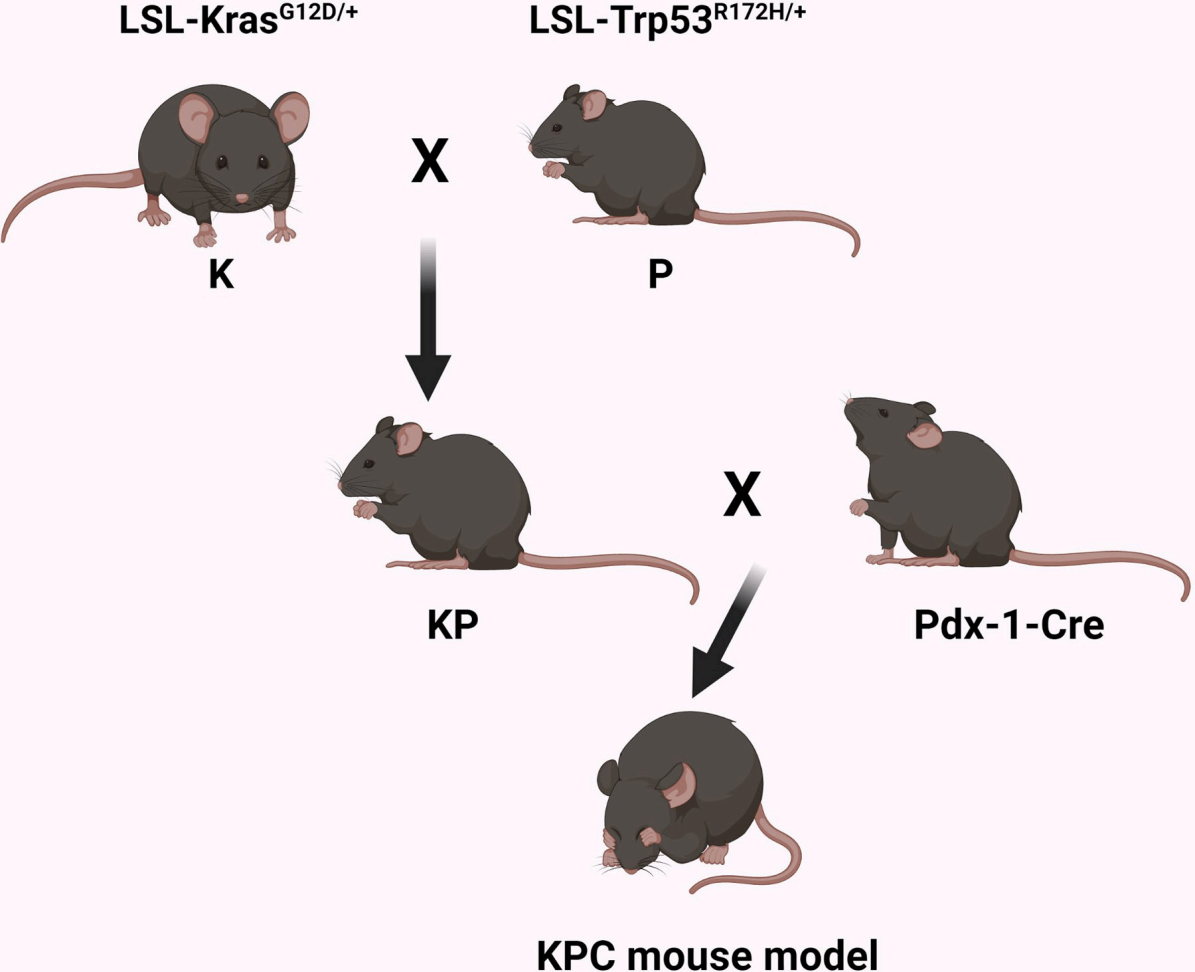
Generation of KPC mice. KPC mice are generated by crossing K, P, and Pdx-1-Cre mice. K mice, activating point mutation (G12D) in K-Ras gene, P-mice, dominant mutation (R172H) in P53 tumor suppressor gene, and Pdx-1-Cre, Cre recombinase expressed in the pancreas under the influence of pancreas-specific Pdx-1 promoter.

We generated a preclinical KPC mouse model of PDAC, which recapitulates human PDAC biology, as described earlier [46, 47]. Briefly, we crossed an LSL-Kras^G12D/+^ (K) mouse with an LSL-Trp53^R172H/+^ (P) mouse to generate a KP mouse that was then crossed with Pdx-1-Cre to generate KPC mice (Fig 1). The Pdx-1-Cre expressed Cre recombinase protein in pancreatic cells under the expression of the pancreas-specific Pdx-1 promoter.

The maintenance of KPC mice is challenging as their lifespan is short (average 10–20 weeks) due to fast tumor growth and progression [47]. Therefore, we considered 10–15 weeks of tumor-bearing in this study. K-, P-, and Pdx-1-Cre mice were obtained for the National Institute of Health (NIH), USA. All studies performed on mice were approved by the Animal Care and Use Committee (IACUC) of Kansas City VA hospital facilities per NIH guidelines for the care and use of laboratory animals.

For endpoint detection, behavioral analyses were critically performed. These included weight loss, hair loss, lack of movement due to perineural invasion (PNI) pain, and food consumption.

### Polymer synthesis of diblock / PEG conjugated pH-responsive diblock polymer synthesis

The synthesis of the pH-responsive diblock copolymers has been described earlier [40, 48, 49]. Briefly, pentaflourophenol and bis (methoxy propionic acid) was used to synthesize the polymer precursor PEG-b-poly(carbonate) via a ring-opening polymerization reactions [50]. The synthesized copolymer was further modified with 2-pyrrolidine-1- yl-ethyl-amine (pka = 5.4) to render the macromolecule pH-responsive [40]. Finally, the synthesized block copolymer, abbreviated as PEG-py, was primarily characterized by ^1^H NMR and FTIR spectroscopy **(**S1 Fig**)**.

### Nanoparticle preparation

The hydrophobic drug ERKi was nano-encapsulated within the pH-responsive block copolymer PEG-py by the nanoprecipitation method following our earlier published protocols. The copolymer (PEG-py) and ERKi were dissolved in DMSO and added to PBS buffer (pH-7.4) dropwise under stirring conditions. The nanoparticles were further dialyzed using a float-a-layer (with molecular weight cutoff of 12k g/mol) followed by filtration through a 2μm PES filter to remove unencapsulated drugs. Characterization of drug-loaded nanoparticles has been carried out following our earlier reports [48]. The filtered nanoparticle suspension was characterized by measuring the particle size and surface charge (zeta potential) using dynamic light scattering (DLS) experiments. Alexa Fluor-647 was co-encapsulated within the block copolymer during the nanoprecipitation method to prepare the dye-loaded nanoparticles. For the preparation of dye-conjugated trafficking nanoformulation, Alexa Fluor 647 dye was conjugated within the copolymeric nanoformulation following the methods described earlier [51].

### Preparation of iRGD conjugated nanoparticulate formulation

As described earlier, the iRGD peptide was conjugated with a pH-responsive block copolymer using "click" chemistry [52] (S1 Fig). iRGD conjugated copolymers were purified via dialysis (MWCO 1000 Da) for 72h.

### Drug release study of ERKi nanoparticles

The amount of drug (ERKi) released from pH-responsive nanoparticles was measured by a dialysis-based method using a Float-a = Lyzer (MWCO 3.5–5 kDa). Briefly, 1 ml of the nanoformulation was placed inside the dialysis chamber. Next, the formulation was dialyzed against 5 ml of PBS buffer, either in the presence or absence of 10% lysis buffer (1 mM EDTA 1 mM EGTA, 1 mM NaF, 20 mM Na_4_P_2_O_7_, 2 mM Na_3_VO_4_, 1% Triton X-100, 10% glycerol, 0.1% SDS, 0.5% deoxycholate. This buffer has been procured from Sigma Aldrich and the pH was adjusted to the desired range. The commercial buffer was further reinforced by 10-unit carboxypeptidase A [cathepsin A]. The objective of this buffer composition is to mimic the microenvironment of lysosomes.

Next, the amount of drug released at different time points was measured by withdrawing 1ml of fluid from the bulk environment outside the dialysis chamber. Finally, the same volume of respective fresh medium was replaced to maintain the overall bulk volume constant.

### Flow cytometric analysis

MIA PaCa-2 cells were seeded in a six-well cell culture plate and treated with Alexa Fluor 647-loaded, iRGD conjugated nanoparticles for 6 and 12h, except for the control. After trypsinization and washing with PBS, the cells were finally suspended in PBS (pH 7.4). The percentage of labeled nanoparticle uptake by the cancer cells was analyzed using a BD Accuri C6 Flow Biosciences Cytometer. All experiments were conducted in eight sets of experiments.

### Confocal fluorescence microscopy

MIA PaCa-2 cells were plated at 5000 cells / well in ibidi® glass bottom dishes (35 mm) and allowed to grow to about 70 percent confluency. The cells were then incubated with dye (Alexa Fluorine 647) loaded iRGD conjugated nanoparticles for six h and 12h, except for the control group (0h). Next, the cells were washed with 1xPBS and imaged using a Zeiss AxioObserver Z1 microscope equipped with an LSM700 laser scanning module (Zeiss, Thornwood, NY) at 40X magnification with a 40x/1.3 Plan-Apochromat lens.

### Clonogenic survival assay

The clonogenic survival or colony formation assay was performed using the previously described protocol [53, 54]. Briefly, untreated and treated MIA PaCa-2 cells were seeded in a six-well plate at 400 cells per well. After 14 days, cells were fixed and stained with a crystal violet solution. The colonies were then counted using a colony Dot-it imaging station (UVP).

### The synergy assay of drug combinations

The PDAC cells (5000 cells/well) were seeded in 96-well plates. The ~60% confluent cells were then treated with the different doses of free or the nanoparticle encapsulated form of SCH 772984 (0–100 nM), GEM (0–1000 nM), and their combination doses for 72 h. The synergy assay was performed as described earlier using Combenefit® software [40].

### In vitro migration

*In vitro* migration of PDAC cells was performed as described previously [54]. Briefly, untreated and treated PDAC cells (10,000 cells/well of twelve-well plates) were seeded into the top chamber insert containing serum-free media. The DMEM containing 10% FBS was added to the bottom chamber as a chemoattractant [54]. After 24h incubation in the CO_2_ incubator, the migrated cells were attached to the outer surface of the insert stained with crystal violet solution, and migrated cells were photographed using a Leica inverted microscope. The membranes were then solubilized with 10% acetic acid, and optical density (OD) was measured using a microplate reader at 450 nm. We examined three wells for each experimental condition and repeated the experiments three times. All studies performed on mice were approved by the Animal Care and Use Committee (IACUC) of Kansas City VA hospital facilities per NIH guidelines for the care and use of laboratory animals.

### Wound healing assay

The wound healing or scratch assay was performed as previously described [55]. Briefly, the MIA PaCa-2 cells (untreated and untreated) were plated into each well of the six-well plate. Cells were allowed to grow to get a confluent monolayered cell. A scratch was made in the confluent cell layer, and the images were captured. The cells were then treated with drugs for 72h. Cell motilities were measured, images were taken, and the wound healing rate was calculated as described earlier [56, 57].

### Western blot analysis

The Western blot analysis was the same as described earlier [40, 54]—briefly, MIA PaCa-2 cells were lysed by RIPA buffer (Cell Signaling Technology), and protein concentration was measured. Then, each sample’s 50 μg protein was loaded, and SDS-PAGE gel electrophoresis was performed, followed by semi-dry transfer into Nitrocellulose membrane using a Trans-blot Turbo Transfer System (Bio-Rad). Finally, membranes were probed with mouse antibodies against neuropilin-1 (NRP-1), CCN1, CCN2, SHh, and β-actin (Cell Signaling Technology and Santa Cruz), treated with peroxidase-conjugated goat anti-mouse secondary antibody, and then visualized by enhanced chemiluminescence.

### *In vivo* treatment

The animal studies were carried out strictly in the Guide for the Care and Use of Laboratory Animals of the National Institutes of Health. The protocol was conducted with the approval of the Animal Ethics Committee of the Kansas Coty VA Medical Center, Kansas City, MO (protocol No. SNB003). All surgery was performed under isoflurane anesthesia, and all efforts were made to minimize suffering. The tumor-bearing KPC mice of different ages (8–10 weeks old), confirmed by ultrasound imaging as described by us [58], were randomized to the respective treatment groups (n = 5). This study’s initial average tumor size was 100 mm^3^ ± 5 mm^3^ volume. Free GEM (40mg/Kg), iRGD-conjugated, pH-responsive nanoparticles (abbreviated as iRGD-pHNPs-ERKi, 75 mg/kg for SCH772984), or a combination of these two drugs were administrated intraperitoneally (i.p.) twice a week for four weeks. Weekly tumor growth/volume measurement is challenging in the KPC autochthonous mouse model. Thus, the tumors were collected, and wet tumor weight was measured after the termination of the experiment or when the signs of morbidity (endpoint) were recognized. These include abnormal posture, rough hair coat, head tucked into the abdomen, and body weight loss (20% loss over the few days). PBS and NP alone were used as controls. We used a human equivalent dose of each drug for this study. All mice were humanly euthanized by CO_2_ asphyxiation, and tumors were excised, wet weight measured, and collected for histologic analysis.

### Statistical analysis

The statistical analysis was performed using Graph Pad Prism 8(GraphPad Software, Inc., La Jolla, CA, USA) and PASS15softwares, NCSS, LLC (Kaysville, UT, USA). Unless stated otherwise, the results are presented as mean ± standard deviation (SD). Means between the groups were calculated and compared among or within the variables using a two-sided Student’s t-test. *In vitro* migration and motility of PDAC cells were determined using a two-sided Student’s t-test and two-way ANOVA. A P-value of 0.05 was considered statistically significant. We calculated the required sample size for in vitro experiments using the previously established method [59], and n = 5–8 cultures per group and time point, assuming a comparison-wise type I error of 5% and power of 80% to detect the probability of concordance of 75%. The required number of mice indicated at least five per group, and the time point, assuming the power of 85%, type I error of 5%, probability of concordance between treatment and tumor measurements of 75%, and experimental success rate of 80%.

A linear regression model of tumor weight was fitted after four weeks of treatment. The model included the mice treated with ERKi-loaded nanoparticles (ERKi-NPs, N = 5), free GEM (N = 5), ERKi+GEM (N = 5), or saline injections (control group, N = 5) (S1 Table). Three dummy variables were used as the model-independent variables to represent the four groups. Further, we used the Kaplan-Meier estimator to plot survival. The entire study was performed blindly by two or more investigators.

## Results

### Morphology and stability study of iRGD conjugated pH-responsive polymer encapsulated ERKi (^iRGD-pH^NPs-ERKi)

**Fig 2:**
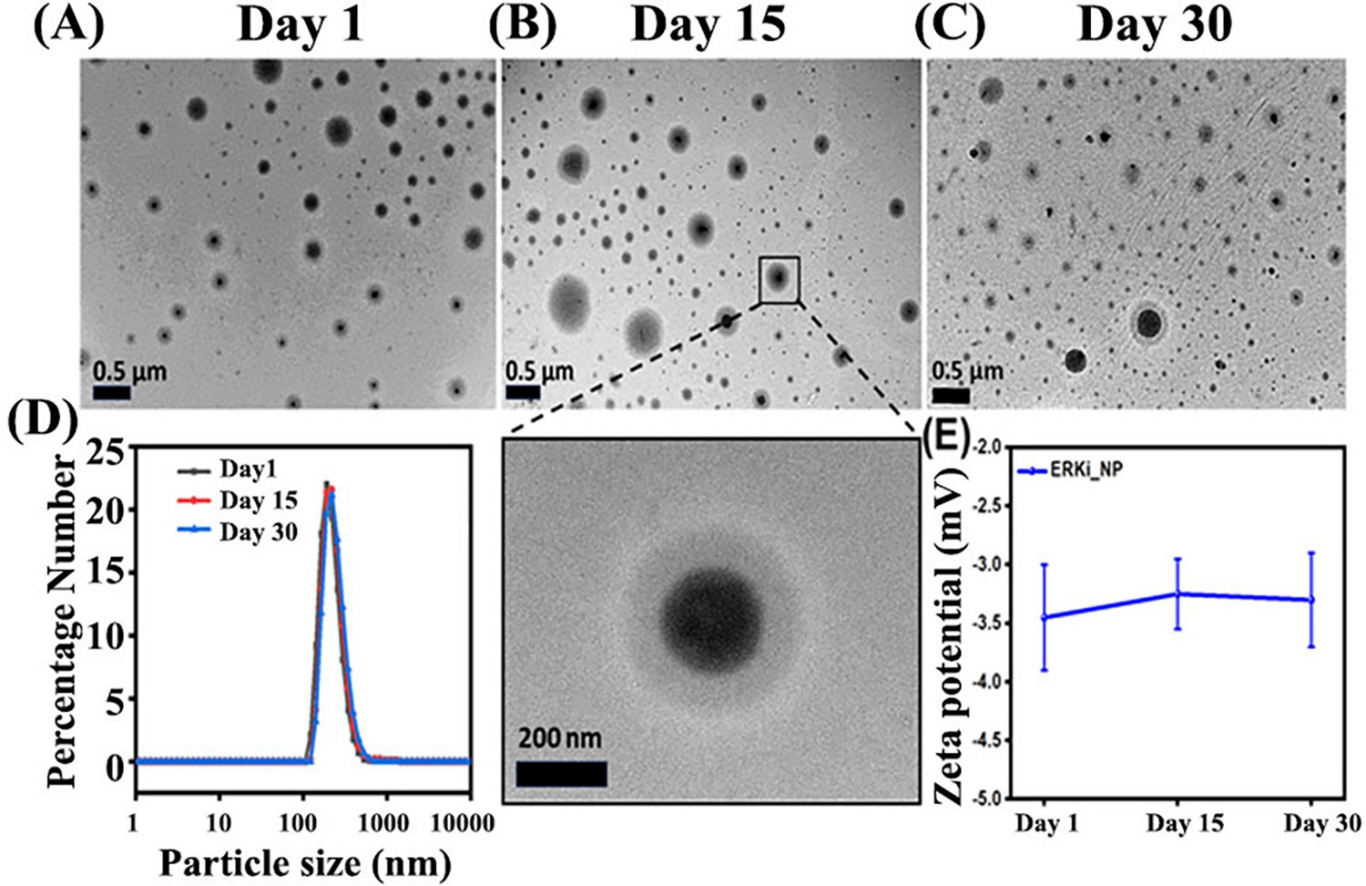
Quality controls (QC) of nanoparticles. TEM micrograph detecting particle size of PEG-Py nanoparticles at **(A)** Day 1, **(B)** Day 15, and **(C)** Day 30. **(D)**. The DLS study measured the particle size distribution range for Day 1, Day 15, and Day 30 to validate TEM images. **(E)**. Variation of zeta potential of PEG-Py for Day 1, Day 15, and Day 30 to demonstrate the stability of nanoparticle throughout the time.

To investigate the morphology and stability of the nanoparticles, we examined the particle size and surface charge of PEG-py nanoparticles by dynamic light scattering representing the particles’ hydrodynamic diameter. Additionally, the particle size in lyophilized form and their surface morphology were confirmed using Transmission electron microscopy (TEM). TEM images showed nanoparticles demonstrated (Fig 2) a smooth surface and spherical shape.

Furthermore, we confirmed the stability of the nanoparticles at 4°C refrigerated conditions by measuring TEM, size distribution, and zeta potential at different time points over a month after synthesis. According to the TEM results, particle size was found to be 191±19nm (Fig 2A), 204±24nm (Fig 2B), and 205±22 nm (Fig 2C) after Day 1, Day 15, and Day 30 post-synthesis, respectively. Hydrodynamic diameters of particles measured via DLS were 255±17 nm for Day 1, 269±18nm for Day 15, and 271±21 nm for Day 30 (Fig 2D) post-synthesis. The variation in the particle size was observed in TEM and DLS data due to the dried and hydrated form of the samples used for these studies. This result suggested that the nanoparticles were stable for at least 30 days post-synthesis under room conditions.

### Drug release and kinetic study of ^iRGD-pH^NPs under an acidic or hypoxic microenvironment

**Fig 3:**
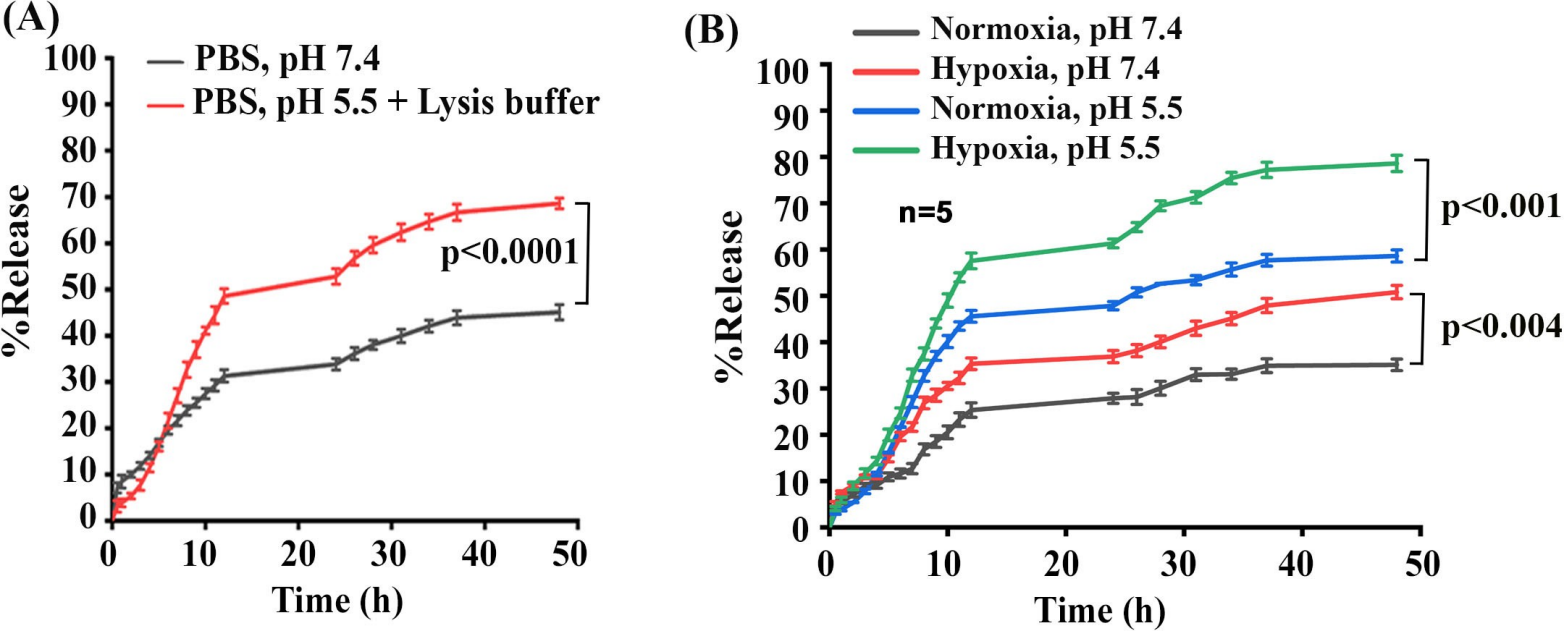
Drug-release profiles in different environments. **(A)**. Cumulative drug release profile in PBS buffer and PBS with 10% added Lysis buffer solution, and **(B).** Effect of hypoxia and low pH on ERKi release from ^iRGD-pH^NPs in a cell-free system.

Our previous studies have shown that ERKi-loaded ^pH^NPs released <20% of encapsulated ERKi in physiological pH (7.4). However, when placed at pH 5.5, the ^pH^NPs released >50% ERKi in 24h. NPs without the pH-responsive linker failed to release a significant amount of drugs under acidic conditions [40]. Therefore, we first investigated the Drug (ERKi) release capacity of ^iRGD-pH^NPs in phosphate buffer saline (PBS, pH 7.2) or PBS with a 10% lysis buffer solution (pH 5.5) containing lysosomal enzymes (Fig 3A). We found that in PBS, 31% cumulative drug was released within 12 h followed by additional 13% cumulative drug release within the next 36 h. In contrast, in PBS with a 10% lysis buffer solution, we found 49% cumulative drug released over 12 h, followed by an additional 20% cumulative drug released over the next 36 h. Both release profiles demonstrated that the drug release rate was significant for the first 12 h, followed by a decrease in release rate over the next 36 h to reach saturation. Moreover, in the presence of a 10% lysis buffer solution with acidic pH, the PEG-py polymer-drug release rate from nanoparticles was enhanced by ~18%, indicating lysosomal autophagic degradation [60] is implicated in drug-releasing from ^iRGD-pH^NPs.

One of the unique features of the solid tumor microenvironment is acidic, mainly due to hypoxia [61]. Thus, we sought to test if ^iRGD-pH^NPs effectively release drugs in a hypoxic environment. The cell-free studies found that ^iRGD-pH^NPs can effectively release ERKi in hypoxic conditions with or without lowering the pH. However, hypoxia with low pH exhibited a significant release of ERKi compared to other conditions (Fig 3B).

### Cellular uptake and internalization of the ^iRGD-pH^NPS-ERKi in MIA-PaCa-2 cells

**Fig 4:**
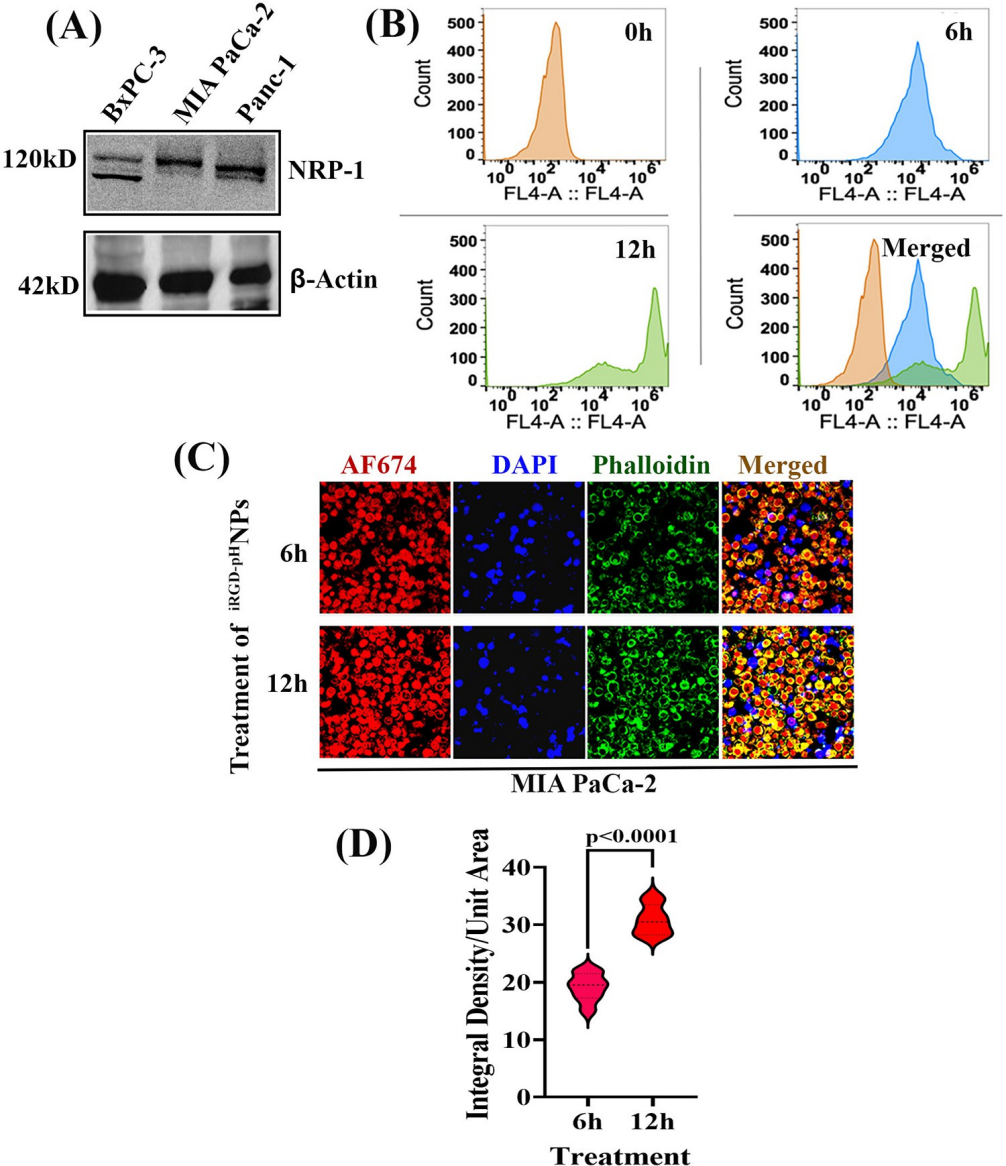
Cellular uptake study of Alexa Fluor 647 (AF 647) labeled iRGD tagged pH-responsive nanoparticles on MIA PaCa-2 cells. **(A).** NRP-1 status in PDAC cell lines, **(B).** Quantitative determination of fluorescently labeled Mia PaCa-2 cells using FACs analysis after 0h, 12 h, and 24 h of incubation with AF 647-labeled nanoparticles. **(C).** Cells were treated with dye-labeled nanoparticles for six h and 12h, and cellular distribution was determined using confocal microscopy and **(D).** Violin diagram representing the integral density/ Unit Area of AF 647 labeled nanoparticles, DAPI (blue), and Phalloidin (green) used for the detection nucleus and cytoplasm of a cell. Data presented as mean ± SD, n = 8.

The cellular uptake of the Alexa Fluor 647 labeled iRGD tagged pH-responsive nanoparticles was investigated in MIA PaCa-2 cells. It was reported that the iRGD peptide (sequence: CRGDKGPDC) binds with the cell surface co-receptor of neuropilin 1 (NRP1) through binding with α_v_ integrins and unmasking the CendR motif (R/KXXR/ K). This sequential process promotes cell penetration via rapid endocytosis/macropinocytosis [62–66]. We, therefore, first investigated the binding affinity of iRGD decorated pH-responsive nanoparticles in MIA PaCa-2 cells that have been found to overexpress NRP-1 (Fig 4A, S2 Fig) [67]. To do so, cells were exposed to AF647 labeled NPs for 0 h, 06 h, and 12 h, and cellular uptake of fluorescently labeled NPs was quantified using FACs analysis. We found a time-dependent increase in AF647-labeled cell numbers (Fig 4B). Next, we investigated cellular uptake and internalization of AF647-labeled NPs (Red color) in MIA PaCa-2 cells using confocal microscopic imaging following incubation with the NPs for 6 and 12 h. Results indicated that the cellular uptake of nanoparticles by MIA PaCa-2 cells was increased significantly in a time-dependent fashion (Fig 4C & 4D). The studies also indicated that the NPs were localized in the cytosol and the nucleus.

### ^iRGD-pH^NPS-ERKi and free GEM are synergistically cytotoxic in PDAC cells

**Fig 5:**
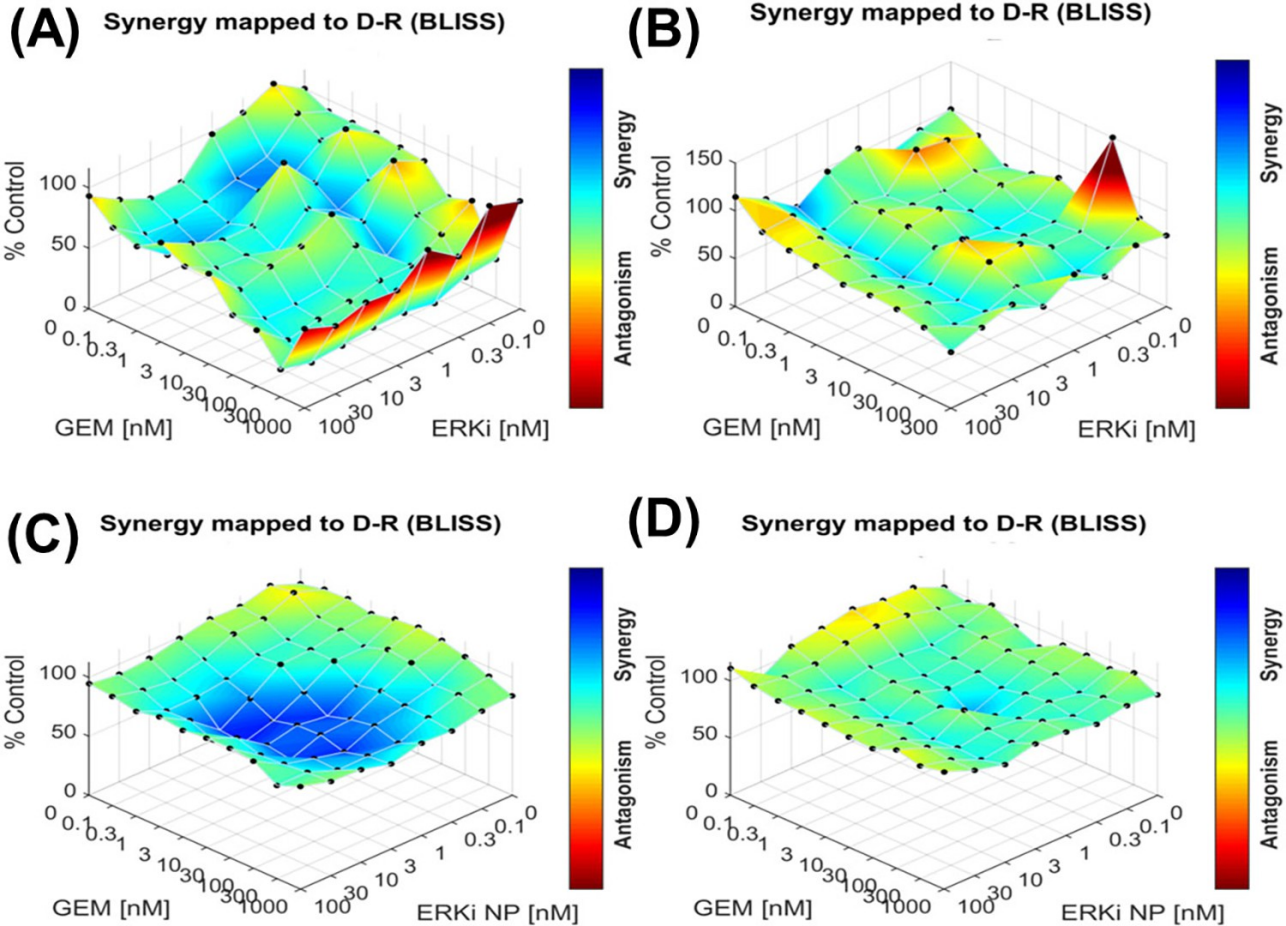
Dose-response synergy map of different drug combinations using Bliss synergy model. **(A).** Free ERKi and Free Gem on MIA PaCa-2 cells, **(B).** Free ERKi and Free GEM on Panc1, **(C).** ERKi NP and free GEM on MIA PaCa-2 and **(D).** ERKi NP and free GEM on Panc1.

Earlier studies have reported that combining two or more therapeutic agents is the cornerstone of cancer therapy as they show an additive or synergistic effect to target cancer-promoting or cancer-sustaining pathways [68]. Recently, we have shown that encapsulated ERKi and GEM in pH-responsive nanoparticles exhibit a synergistic cytotoxic effect on PDAC cells [40]. Therefore, this study sought to investigate whether ^iRGD-pH^NPS-ERKi and free GEM combination exhibit a synergistic cytotoxic effect on PDAC cells. To test the hypothesis, the combined cytotoxicity of ERKi (SCH 772984)-^iRGD-pH^NPs with free GEM was investigated in MIA PaCa-2 and Panc-1 cell lines by a surface plot based on the percent excess of the Bliss prediction using the average response measures at each combination dose [69]. We found an impressive synergistic cytotoxic effect in MIA PaCa-2 cells after 72 h incubation with drugs (Fig 5). In contrast, the synergistic interaction of these drugs’ combination on Panc1 cells was not notable as the MIA PaCa-2 cell line. On the contrary, free-ERKi and GEM exhibit weak or no synergistic effects. Although the mechanism is unclear, the results indicated that the combination of free GEM and encapsulated ERKi synergized when used to suppress the growth and proliferation of MIA-PaCa-2 and Panc-1 cells. Thus, we mainly used MIA PaCa-2 cells for physiological studies.

### ERKi-^iRGD-pH^NPs and free GEM alone or in combination impair the Colony-forming ability of PDAC cells

**Fig 6:**
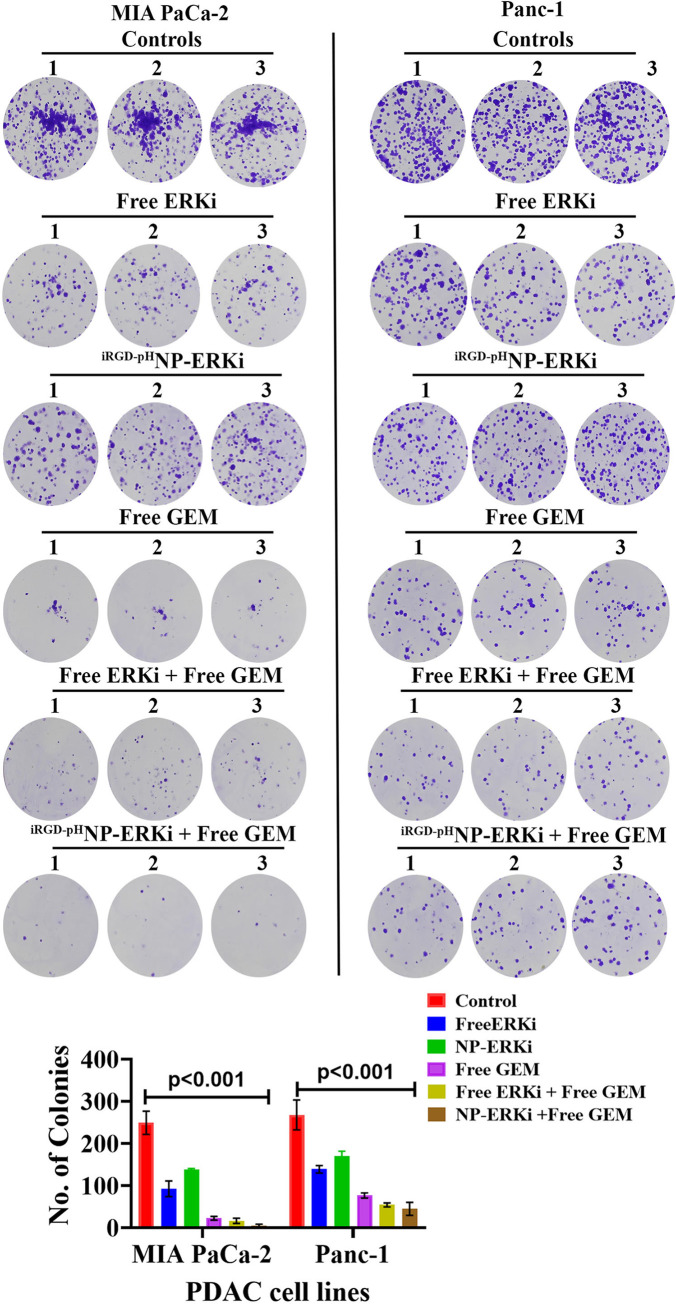
Clonogenic survival assay on MIA PaCa-2 and Panc-1 cells using a different combination of free GEM, Free ERK inhibitor, and nano encapsulated ERK inhibitor. Cells were treated with 0.1% DMSO control, free GEM, free ERKi, ERKi nanoparticles, or a combination of drugs for 72h. Cells (400 cells) were added to each well of the six-well plate for 14 days for colony formation. Colonies were counted using the colony Dot-it imaging station. The bar graph represents the mean ± SD of three experiments.

The *in vitro* colony-forming ability indicates clonal cell growth, thus a hallmark of aggressive cancer cells or cancer stem cells. The ERK1/2-signaling plays a vital role in cancer cell growth and colony formation [70]. This study, to test the functional efficacy of ERKi-^iRGD-pH^NPs, thus aimed to investigate whether ERKi-^iRGD-pH^NPs can reduce the colony-forming ability of PDAC cells and exhibit an additive impact in the presence of free GEM. We analyzed the colony formation capacity of MIA PaCa-2 and PANC-1 cells after treating the cells with the IC_50_ concentration of free GEM, nano-encapsulated ERKi and combining both drugs. We found a significant inhibition of the colony formation in the combined treatment of free GEM along with nano-encapsulated ERKi compared to the treatment involving GEM alone, encapsulated ERKi, free GEM, and ERKi combination, or the untreated control (Fig 6).

### In vitro migration of pancreatic cancer cells was decreased by combination therapy

**Fig 7:**
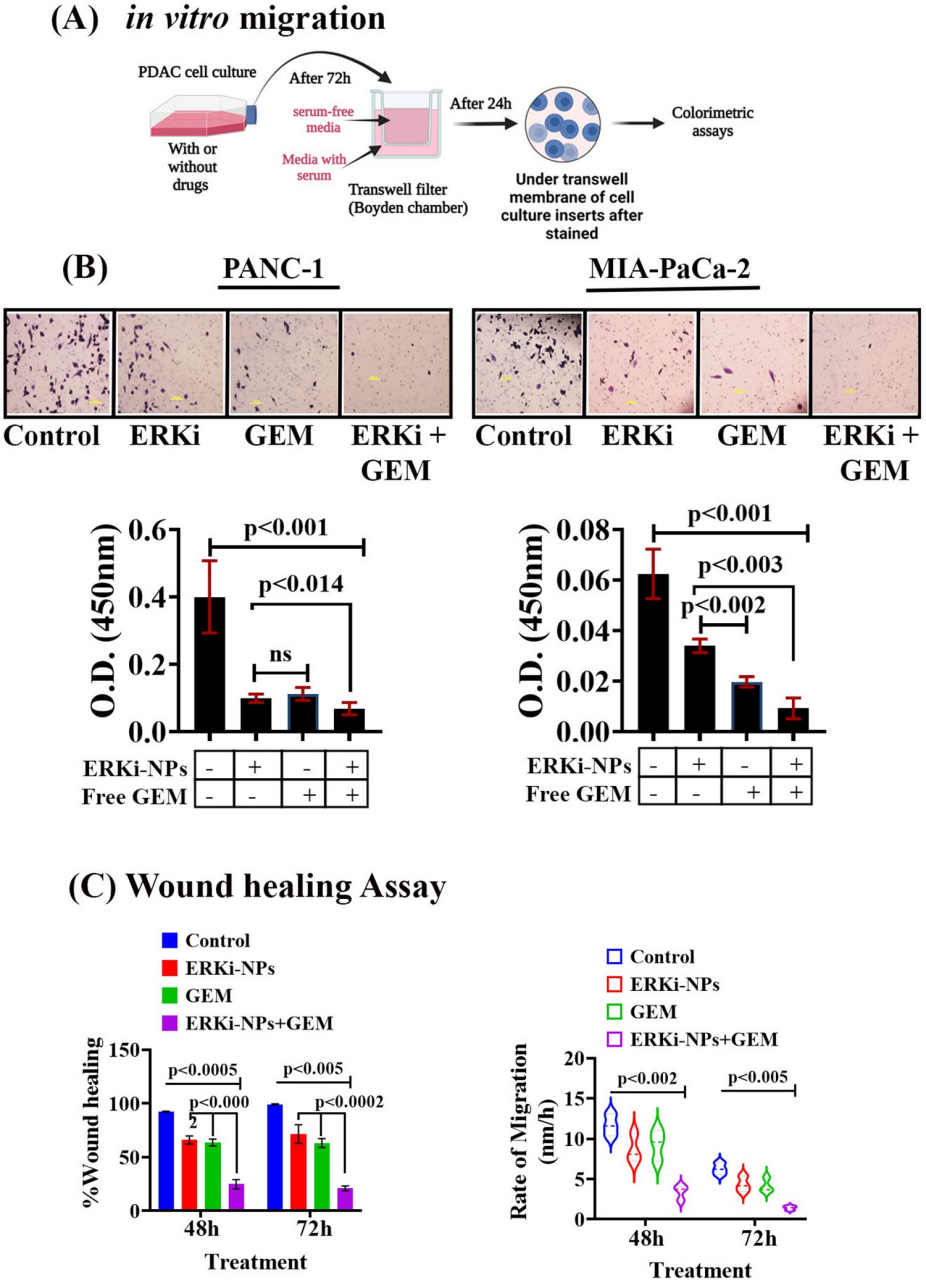
Effect of ERKi-NPs on invasive phenotypes of PDAC cells in the presence or absence of free gemcitabine. **(A).** The proposed experimental strategy of *in vitro* migration, **(B).** Representative microphotograph (10x, crystal violet staining) and quantification of Transwell migration assay of PDAC cell lines with or without treatment groups, and **(C).** wound-healing assay in untreated and treated groups of MIA PaCa-2 cells. Data represent means ± SD of three independent experiments. The p values were calculated using one-way ANOVA and two-tailed unpaired Student’s t-test. ns = not significant.

The RAS-ERK pathway is conserved in promoting cancer cell migration and invasion [71]. Therefore, to test the additional functional potency of ^iRGD-pH^NPs-ERKi, we conducted an *in vitro* migration assay and a wound healing assay to determine the cancer cell migration ability after the drug treatment. We found that free GEM or encapsulated ERKi decreased PDAC cell migration. However, the encapsulated ERKi and GEM combination significantly impacted cell mobility at 48 and 72h (Fig 7).

### Combination treatment of ERKi- ^iRGD-pH^NPs and GEM impairs K-Ras-dependent PDAC growth in an autochthonous mouse model

**Fig 8:**
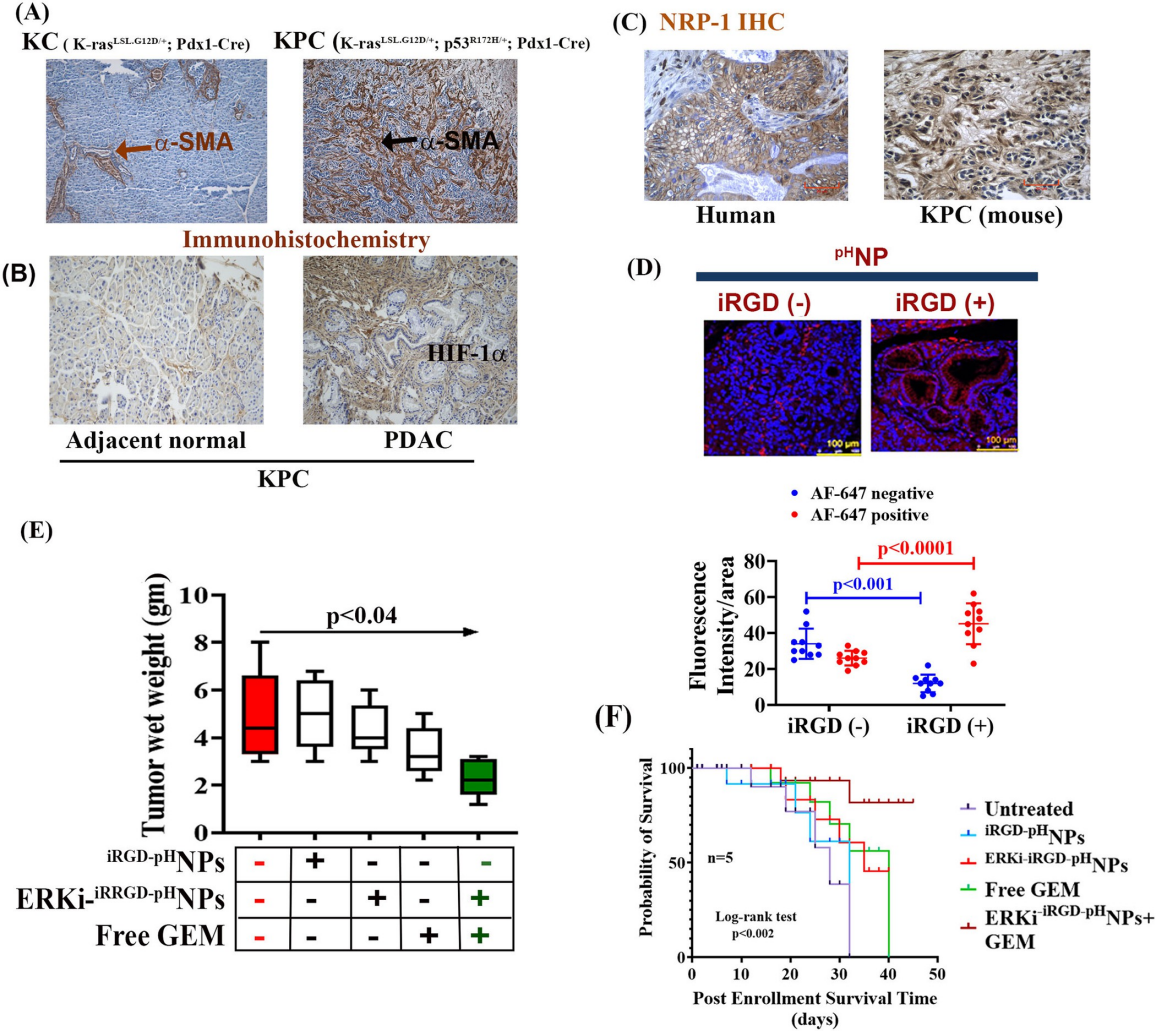
The treatment effect of ERKi-^iRGD-pH-^NPs and GEM on PDAC tumor growth in the KPC mouse model. **(A)**. Detection of desmoplasia by immunohistological evaluation of α-SMA in the KC and KPC mice tumor sections, **(B).** Detection of the hypoxia by Immunohistological evaluation of HIF-1α in a KPC mouse’s adjacent normal and tumor sections. **(C).** Immunohistological evaluation of NRP-1 in the human and KPC mice tumor sections, **(D).** Penetration and distribution of ^pH^NPs with or without iRGD in the PDAC tumors in the KPC model. n, 3 KPC mice. The fluorescent intensity was measured from ten hotspot areas from sections of the tumors of three KPC mice. Scale, 200 μm, Data are mean ± SD. **(E).** GEM treatment in combination with ERKi-^iRGD-pH^NPs showed the minimum tumor growth (blue circles). (n = 5, three males, two females). Data represent mean ±SD. **(F).** Kaplan-Meier analysis depicting cumulative of respective treatment groups.

To evaluate the effect of ERKi-^iRGD-pH^NPs and GEM alone or the combination of these two drugs *in vivo*, we used the K-Ras mutant-dependent PDAC model in KPC mice (Fig 2). Genetically engineered KPC mice recapitulate the pathological characteristics of human pancreatic cancer with desmoplasia [47, 72], comprising a dense fibroinflammatory tumor microenvironment that causes hypoxia and limits drug delivery [73, 74]. Consistent with previous findings, we found tumor-bearing KPC mouse exhibits more desmoplasia than KC mice, as detected by immunostaining of α-SMA, an active stellate cell marker (Fig 8A). Moreover, a hypoxic marker, HIF1α, is markedly elevated in the tumor areas of KPC mice (Fig 8B), suggesting these PDAC tumors with desmoplasia is highly hypoxic.

We next investigated the status of NRP-1 in PDAC in KPC mice and compared it with human PDAC tissue samples. The immunohistochemical studies showed that NRP-1 is overexpressed in human and mouse PDAC samples (Fig 8C), indicating that the KPC PDAC mouse model is ideal for iRGD-mediated drug delivery studies.

We then evaluated the penetration of ^iRGD-pH^NPs in desmoplastic PDAC tumors in KPC mice, followed by the accumulation of NPs in the tumors. For imaging, we incorporated an Alexa Fluor 647 (AF-647) conjugated polymer (5 mol%) in the ^iRGD-pH^NPs and injected them through the tail vein of KPC mice bearing pancreatic tumors (n = 3) for 24h. The tumors were excised and subsequently imaged for fluorescence signals. We observed that the ^iRGD-pH^NPs accumulated significantly in the tumor compared to the non-targeted control (Fig 8D).

Finally, we investigated the effect of ERKi-^iRGDpH^NPs, GEM., or a combination of ERKi-^iRGD-pH^NPs on the chemosensitization of GEM in KPC mice following twice-a-week i.p. injection of saline, ^iRGD-pH^NPs containing 75 mg/kg for SCH772984, free GEM (40 mg/kg for GEM/human equivalent dose), or a combination of ERKi^-iRGD-pH^NPs with GEM into tumor-bearing KPC mice. On average, after four weeks of treatment, tumor weights in mice on GEM alone were 1.20 g smaller than in untreated mice, but this difference was insignificant (p = 0.19, S1 Table). The average difference in tumor weights between mice treated with ERKi NPs alone and untreated mice was insignificant (p = 0.58). In contrast, mice receiving both ERKi NPs and GEM exhibited tumor weights that were 2.56 g smaller than those of untreated mice, a significant difference [p = 0.01; 95% CI, (-4.43, -0.69); S1 Table]. The difference between the effect of the combined therapy and the average of the effects of ERKi NPs and GEM alone was significant [-1.71; p = 0.04; 95% CI, (-3.33,-0.089)], suggesting a statistically significant synergism between ERKi NPs and free GEM (Fig 8E). Furthermore, we found that the combination treatment significantly increased the overall survival of the KPC mice (Fig 8F). Collectively, the effect of ERKi-^iRGD-pH^-NPs or GEM is not remarkable as a combination treatment suggesting ERKi-^iRGD-pH^-NPs and GEM together could be an ideal approach for K-RAS mediated PDAC therapy.

### The expressions of desmoplastic regulatory proteins in PDAC cells are impaired following ERKi treatment

**Fig 9:**
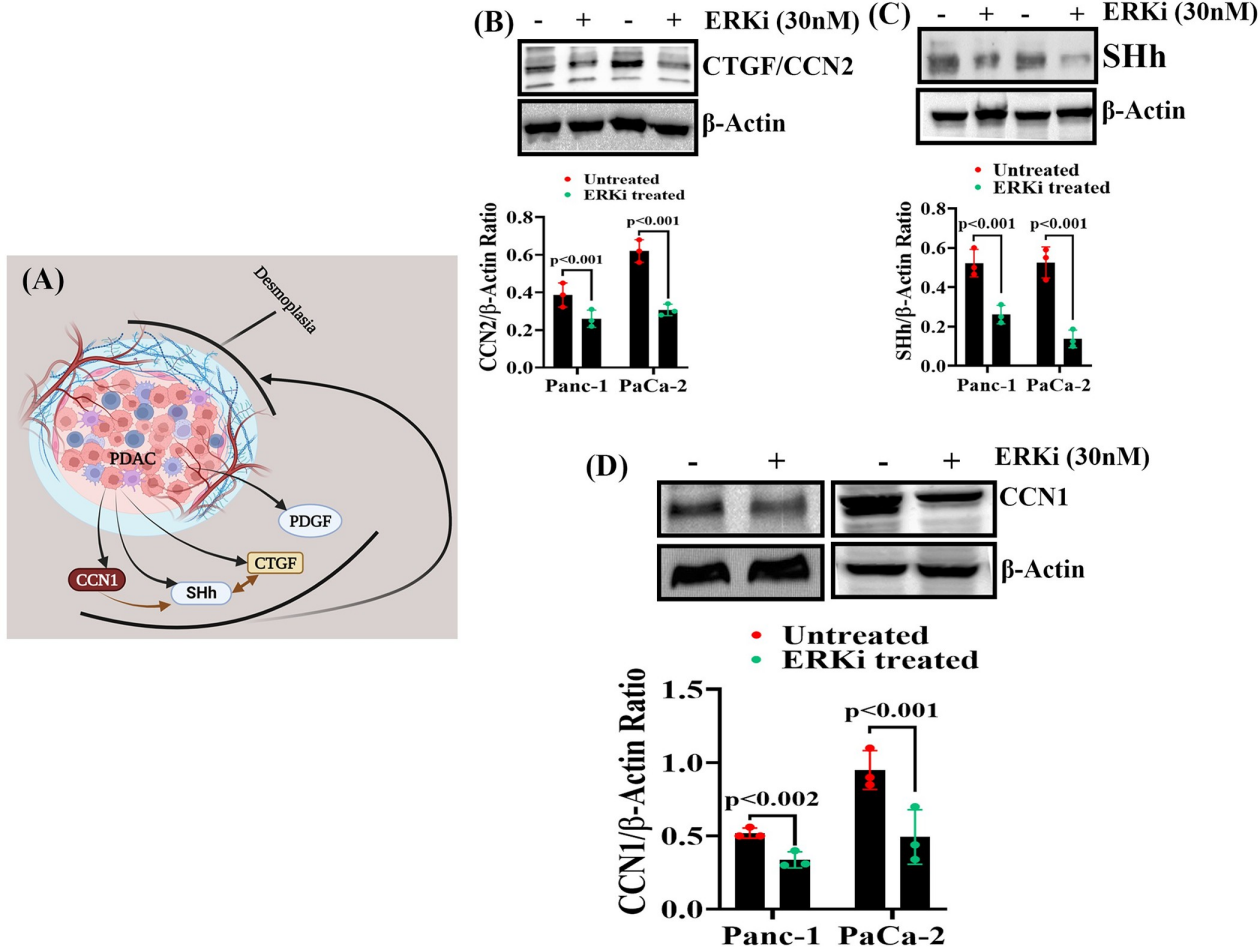
ERKi suppressed the production of desmoplastic regulatory proteins in PDAC cells. **(A).** ERKi targetable desmoplastic regulatory molecules in PDAC. **(B-D).** Immunoblots of lysates from Panc-1 and MIA-PaCa-2 cells treated with ERKi or left untreated. β-Actin antibody was used as a loading control for each immunoblot.

Peritumoral desmoplasia significantly affects intra-tumoral drug delivery [75]. Several secretory proteins, including cell communicating network 1 (CCN1), sonic hedgehog (SHh), and CCN2/CTGF, of PDAC cells help in promoting and maintaining peritumoral desmoplasia by activating myofibroblast cells (Fig 9A) [75–78]. In addition, some of these proteins are intertwined via feed-forward loops. Our study showed that ERKi treatment significantly reduced the production of CCN1, CCN2, and SHh in PDAC cells (Fig 9B–9D) (S2 Fig).

## Discussion

**Fig 10:**
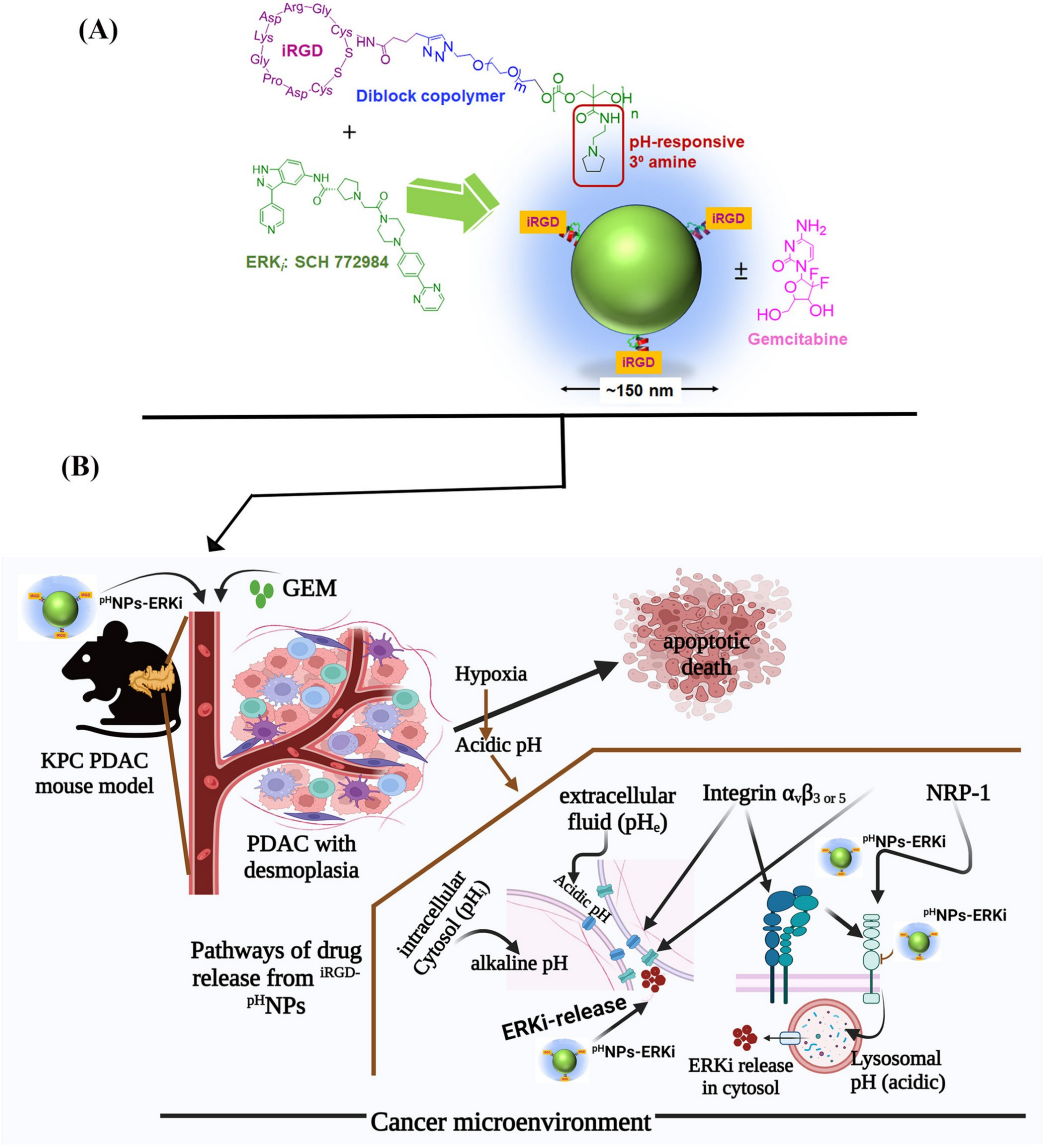
Proposed *in vivo* model of ERKi-^iRGD-pH^NPs drug-releasing pathways in PDAC. The pathways are discussed in the text about the pathways of drug release under hypoxic and low-pH microenvironments. The drawing is adapted from current work and previous findings.

The pH in tumor cells and their microenvironment plays critical roles in the development of the disease and is thus considered an active target for treatment [79]. Previous studies have shown that cancer cells’ intracellular cytosolic pH (pH_i_) is mildly alkaline compared to healthy cells, while cancer cells’ extracellular pH (pH_e_) is acidic. Thus, nanoparticles responsive to the pH gradients are promising for cancer drug delivery [40, 80]. Furthermore, the pH-responsive nanocarriers have therapeutic advantages because, at pH_e_ or lysosomal pH (acidic), they release drugs into the extracellular fluids or cytosol (Fig 10) [80]. The released drugs then destroy cancer cells, and surrounding fibroblast cells form a desmoplastic cage and can be used for cancer immunotherapy [40, 81].

Furthermore, the desmoplastic microenvironment is highly hypoxic, causing lactate formation, and, as a result, pH is decreased in the cancer cells and tumor microenvironment [82]. Thus, low pH or hypoxia in the tumor ecosystem could be the ideal target for releasing drugs using pH-responsive nanocarriers for cancer therapies with no or minimal side effects. Therefore, this study aimed to investigate whether ERKi-loaded pH-responsive nanocarrier grafted with a PDAC-targeted arginine-glycine-aspartic acid (iRGD)-peptide effectively releases ERKi in a pH and hypoxic environment. We demonstrated that ERKi is released in low pH environments, including hypoxic areas, by co-opting multiple mechanisms and showed an additive effect with gemcitabine *in vitro* and *in vivo*. Therefore, as summarized in Fig 10, we propose that the iRGD decorated pH-responsive nanocarriers effectively release drugs in pH_e_, lysosomal pH, and hypoxia zone in PDAC *in vitro* and a KPC mouse model with a hypoxic zone.

Recently, we found that pH-responsive nanocarriers release ERK inhibitors and gemcitabine under cell culture conditions and in the tumors of a subcutaneous xenograft mouse model. As expected from the mechanistic viewpoints, the released drugs significantly impaired *in vitro* cellular proliferation and tumor growth [40] and raised two hypotheses. These include 1. the pH-responsive nanocarriers could be tumor-specific targeted particles if we incorporate iRGD, and 2. Since hypoxia promotes the tumors’ and tumor microenvironment’s pH gradient zones, pH-responsive nanocarriers could release ERKi in hypoxic tumors without further modification.

Our collective studies found pH-responsive ERKi (SCH772984) encapsulated nanoparticles grafted with iRGD peptide, effectively releasing ERKi in hypoxic and low pH environments. They reduced PDAC growth in a KPC autochthonous tumor model in which desmoplastic zones are high. Thus, these studies support the first hypothesis and indicate that iRGD grafted pH-responsive nanoparticles are ideal for delivering drugs into the tumors surrounded by the desmoplastic cage with hypoxic or low pH environments through the possible multiple pathways, as shown in Fig 10. However, further studies are warranted.

Finally, through comprehensive experimental analyses, including colony formation ability and cellular migration as invasive behaviors, and *in vivo* studies, we demonstrated that the therapeutic efficacy of free gemcitabine increased significantly in both *in vitro* and *in vivo* when performed a combination treatment with nanoparticle-encapsulated ERKi. However, *in vivo* system, the effect of a single agent is minimal compared to *in vitro*. In contrast, the combination treatment effect was more pronounced in *vivo*, and gemcitabine efficiency is expected to be weak *vivo* compared to that observed in the tissue culture environment.

ERK inhibitors regulate desmoplastic reactions in the cancer microenvironment [32, 33]. Our *in vitro* and *in vivo* studies found that ERKi significantly impaired the production of several proteins necessary for desmoplasic reaction in PDAC, suggesting destroying desmoplasia might help promote GEM action in these models.

## Conclusion

Our findings have important biological and clinical implications. The pH-responsive nanoparticles used in these studies are rewired chemically and pharmacologically with iRGD tumor penetrating peptide for cancer cell-targeted delivery and releasing ERKi in hypoxic and low pH zones. Finally, the K-RAS-dependent PDAC tumors in genetically engineered KPC mouse models can be sensitized to gemcitabine by combining ERKi treatments. Neither inhibitor has potent single-agent activity in this tumor as in combination. Further, we anticipate that ERKi significantly reduces the production of desmoplastic regulator factors in PDAC cells. For the first time, we showed that an iRGD-tagged pH-responsive nanocarrier would be suitable for off-the-shelf universal use to deliver drugs with challenging PK/PD features to highly hypoxic tumors.

## Supporting information

S1 Table (Related to Fig 8): Linear regression model of tumor weight. (PDF)

S1 Fig (Related to iRGD and NMR). (A-B). 1H NMR spectrum of the polymer and ATR spectra of the iRGD-linked polymer (red) versus starting material (blue).
(PDF)

S2 Fig (Related to Figs 4 and 9). Initial membrane immunoreacted.
(PDF)
