# Supplementary material for: pH-responsive targeted nanoparticles release ERK-inhibitor in the hypoxic zone and sensitize free gemcitabine in mutant K-Ras-addicted pancreatic cancer cells and mouse model
Source: PLoS One. 2024 Apr 30;19(4):e0297749. doi: 10.1371/journal.pone.0297749 (PMC11060587; doi:10.1371/journal.pone.0297749)
Supplement: S1 Table — (PDF) [file pone.0297749.s001.pdf]

Supplementary Information

pH-responsive Targeted nanoparticles release ERK-inhibitor in the hypoxic zone and Sensitize free Gemcitabine in Mutant K-Ras-addicted Pancreatic Cancer Cells and Mouse Model

Debasmita Dutta[1#], Priyanka Ray[1], Archana De[2], Arnab Ghosh[2,3#], Raj Shankar Hazra[1], Pratyusha Ghosh[1,2], Snigdha Banerjee[2,3*], Francisco J.Diaz[4], Sunil P. Upadhyay[2,3], Mohiuddin Quadir[1]* and Sushanta K Banerjee[2,3]*

S1 Table. Linear regression model of tumor weight after four weeks of treatment in mice treated with ERKi NPs, free GEM, ERKi+GEM, or saline injections (control group).

Experimental Condition	Coefficient (grams)[a]	95% CI	P<value
ERKi alone[b]	-0.50	(-2.37, 1.37)	0.58
GEM alone[c]	-1.20	(-3.07, 0.67)	0.19
ERKi+GEM[d]	-2.56	(-4.43, -0.69)	0.01

CI: Confidence interval.

[a]Measures the difference between average tumor weight under the corresponding experimental condition and tumor weight on saline injections.

[b]The dichotomous variable was defined as 1 if the mouse received only ERKi, or 0 otherwise.

[c]The dichotomous variable was defined as 1 if the mouse received only GEM or 0 otherwise.

[d]The dichotomous variable was defined as 1 if the mouse received both ERKi and GEM, or 0 otherwise.
