# Supplementary material for: pH-responsive targeted nanoparticles release ERK-inhibitor in the hypoxic zone and sensitize free gemcitabine in mutant K-Ras-addicted pancreatic cancer cells and mouse model
Source: PLoS One. 2024 Apr 30;19(4):e0297749. doi: 10.1371/journal.pone.0297749 (PMC11060587; doi:10.1371/journal.pone.0297749)
Supplement: S1 Fig — (A-B). 1H NMR spectrum of the polymer and ATR spectra of the iRGD-linked polymer (red) versus starting material (blue). (PDF) [file pone.0297749.s002.pdf]

Supplementary Information

pH-responsive Targeted nanoparticles release ERK-inhibitor in the hypoxic zone and Sensitize free Gemcitabine in Mutant K-Ras-addicted Pancreatic Cancer Cells and Mouse Model

Debasmita Dutta[1#], Priyanka Ray[1], Archana De[2], Arnab Ghosh[2,3#], Raj Shankar Hazra[1], Pratyusha Ghosh[1,2], Snigdha Banerjee[2,3*], Francisco J.Diaz[4], Sunil P. Upadhyay[2,3], Mohiuddin Quadir[1]* and Sushanta K Banerjee[2,3]*

(A) NMR spectrum of mPEG-PY

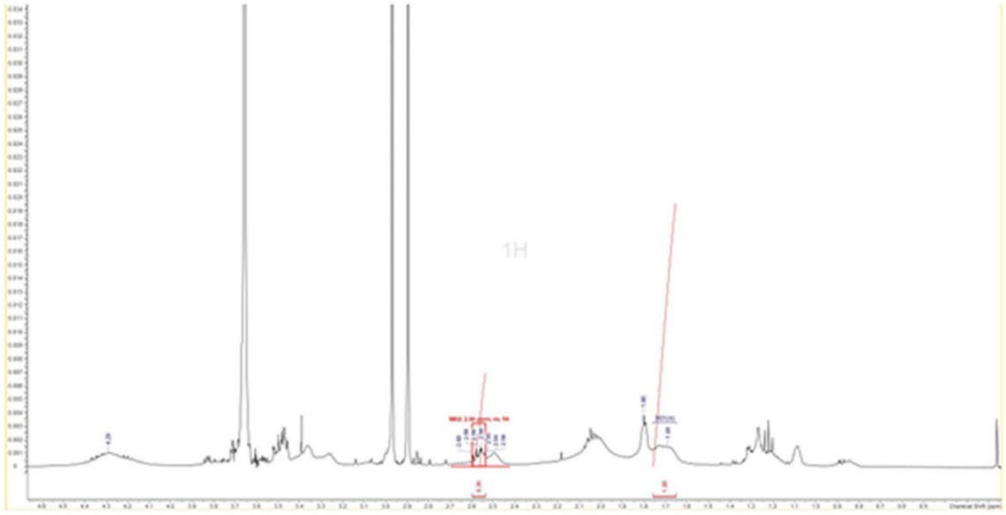

(B) IR spectra of mPEG-OH vs iRGD clicked polymer

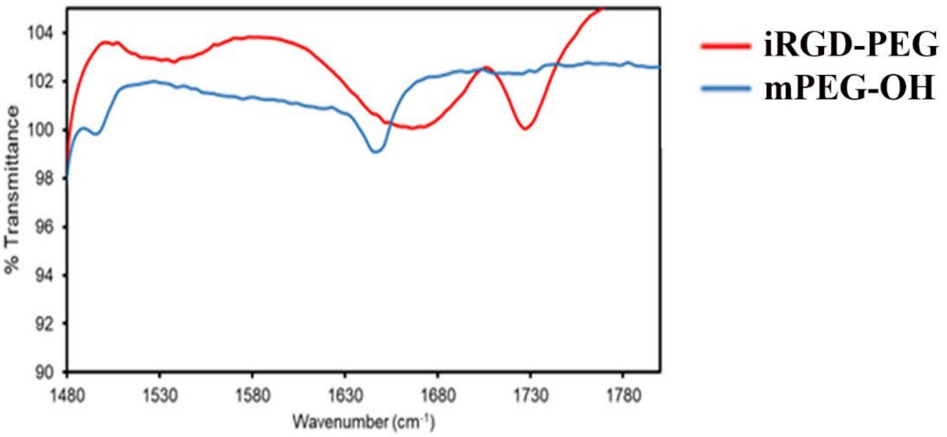

S1 Figure. (A-B). 1H NMR spectrum of the polymer and ATR spectra of the iRGD-linked polymer (red) versus starting material (blue).
