# Supplementary material for: pH-responsive targeted nanoparticles release ERK-inhibitor in the hypoxic zone and sensitize free gemcitabine in mutant K-Ras-addicted pancreatic cancer cells and mouse model
Source: PLoS One. 2024 Apr 30;19(4):e0297749. doi: 10.1371/journal.pone.0297749 (PMC11060587; doi:10.1371/journal.pone.0297749)

Supplementary Information

pH-responsive Targeted nanoparticles release ERK-inhibitor in the hypoxic zone and Sensitize free Gemcitabine in Mutant K-Ras-addicted Pancreatic Cancer Cells and Mouse Model

Debasmita Dutta[1#], Priyanka Ray[1], Archana De[2], Arnab Ghosh[2,3#], Raj Shankar Hazra[1], Pratyusha Ghosh[1,2], Snigdha Banerjee[2,3*], Francisco J.Diaz[4], Sunil P. Upadhyay[2,3], Mohiuddin Quadir[1]* and Sushanta K Banerjee[2,3]*

Uncut Figure 4

Initial membrane immunoreacted with NRP-1 ab

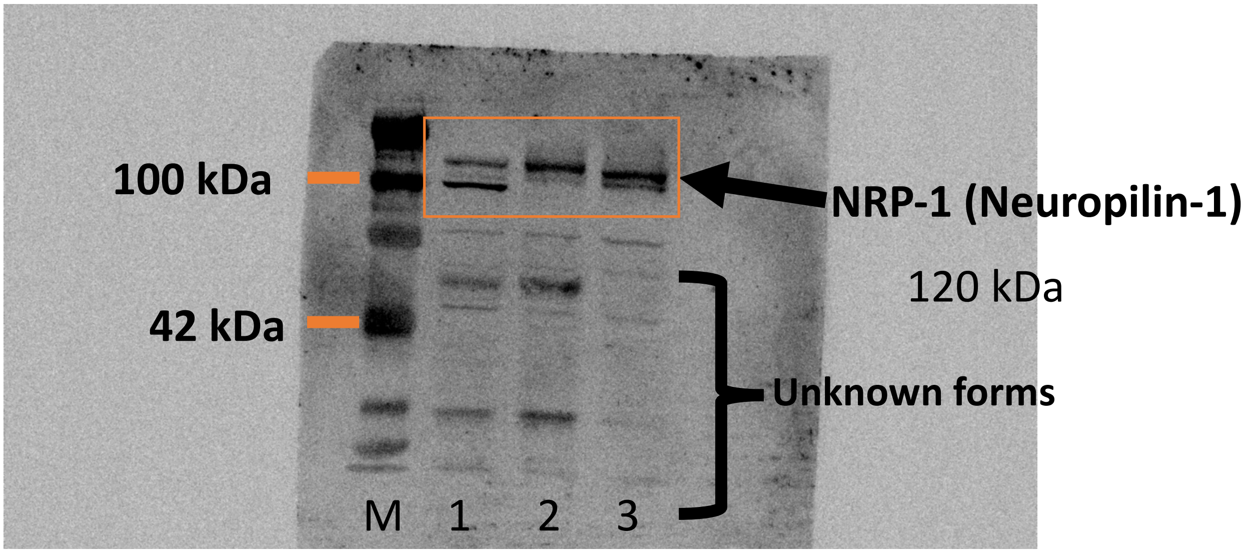

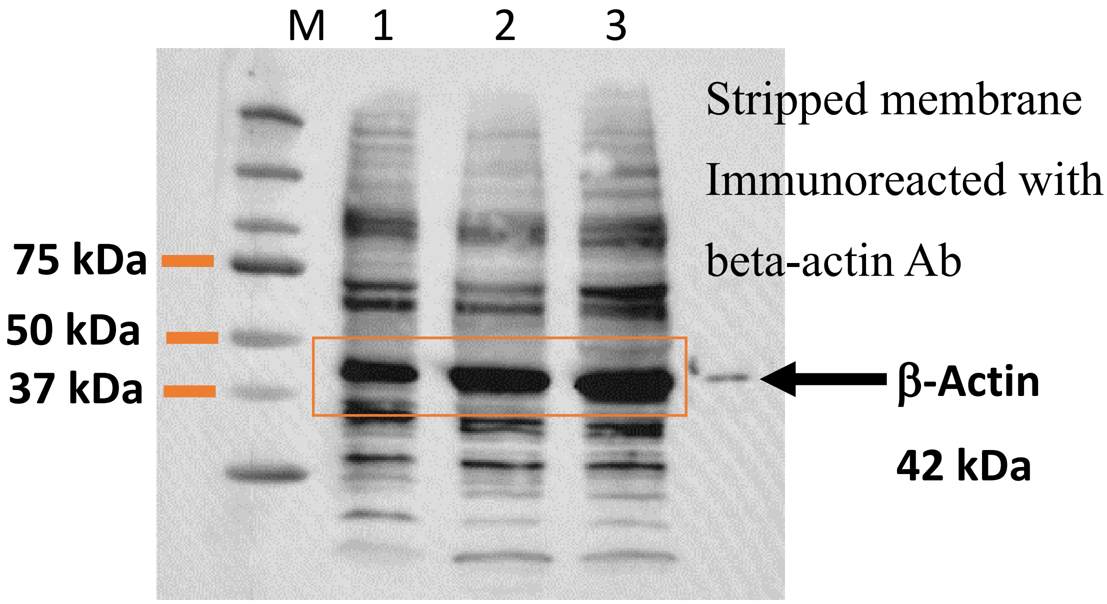

Uncut Figure 9

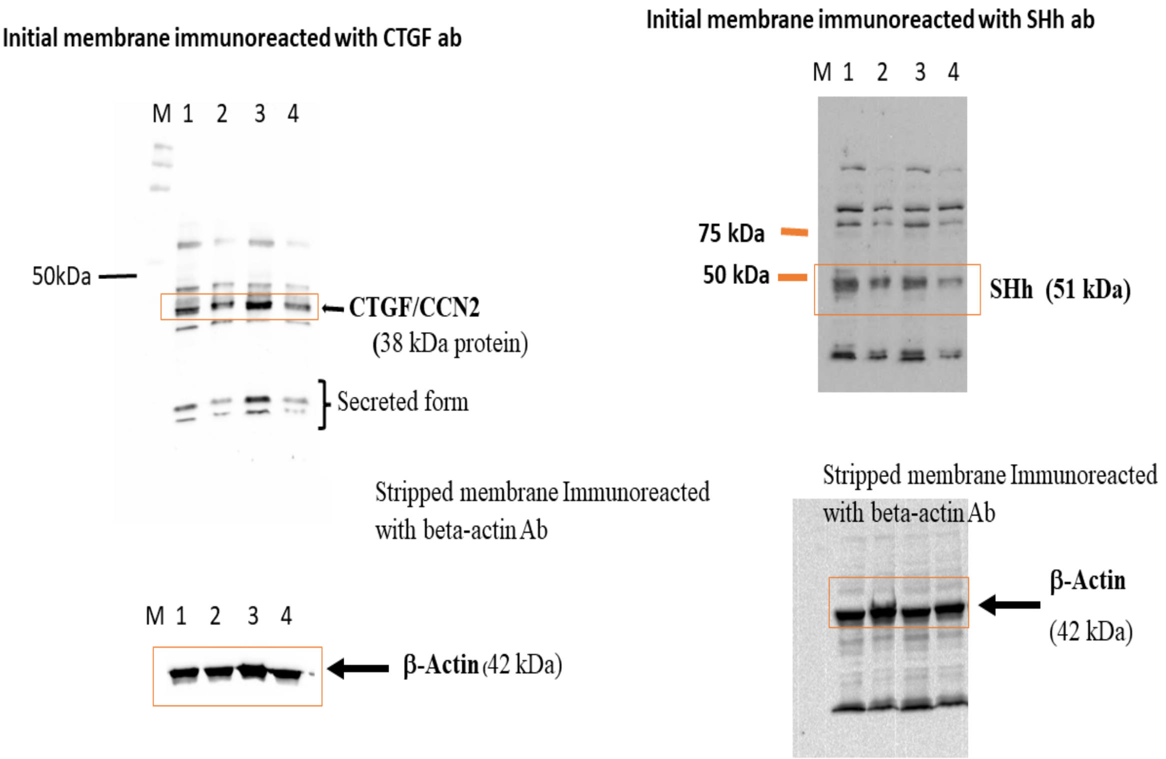

Supplement: S2 Fig — Initial membrane immunoreacted. (PDF) [file pone.0297749.s003.pdf]
